# Multicomponent drug Neurexan mitigates acute stress‐induced insomnia in rats

**DOI:** 10.1111/jsr.13550

**Published:** 2022-01-21

**Authors:** Christopher J. Davis, Michelle A. Schmidt, Kathrin Hemmer, Natascha Krömmelbein, Bernd Seilheimer

**Affiliations:** ^1^ Department of Translational Medicine and Physiology in the Elson S. Floyd College of Medicine and Sleep and Performance Research Center Washington State University Spokane WA USA; ^2^ Heel GmbH Baden‐Baden Germany

**Keywords:** hypnotic, initial/onset insomnia, multitarget, natural drug

## Abstract

The aim of this study was to determine whether the multicomponent drug Neurexan could mitigate acute insomnia after exposure to a psychosocial stressor. We administered Neurexan orally to rats and examined stress‐induced insomnia using the male rat dirty cage exchange method. The neurocircuitry and electrophysiological correlates of the model are characterised, and it represents various human insomnia conditions. Male rats were randomly assigned in a crossover design to six treatment groups and electroencephalography (EEG) electrodes attached. Three groups were exposed to a cage inhabited by another male rat for a week and the other three groups received a clean cage. Prior to cage change, rats were given either no drug, vehicle control or Neurexan. Non‐rapid eye movement (NREM) sleep, REM sleep, and waking were assessed manually via EEG recordings. Group means were compared for sleep latency and for the 2 h after cage change for: time in each state, state‐specific episode duration/frequency, in addition to NREM delta, gamma and REM theta EEG spectral power. Rats administered Neurexan fell asleep faster than vehicle‐treated rats and spent less time awake with shorter, albeit more waking episodes and increased NREM episodes after dirty cage exposure. Neurexan‐treated rats given dirty cages were not statistically different on any outcomes from Neurexan‐treated rats given clean cages, thereby mitigating the stressor. In the EEG power spectra analysed, changes between treatment groups were not detected. This research confirms that Neurexan treatment has somnogenic effects and ameliorates psychological stressor‐induced acute insomnia.

## 1 INTRODUCTION

The demands of fast‐paced lifestyles can lead to decreased health and well‐being, stress‐related symptoms, and in many cases sleep disturbances including insomnia (Åkerstedt, Nordin, Alfredsson, Westerholm, & Kecklund, 2012; Blanco‐Encomienda, García‐Cantero, & Latorre‐Medina, 2020; Ferrie, Kumari, Salo, Singh‐Manoux, & Kivimäki, 2011; Garefelt et al., 2020; Magnusson Hanson et al., 2011). The objective of the present study was to determine the extent to which the natural, multicomponent drug Neurexan (Nx4) could mitigate acute insomnia in rats, resulting from exposure to an acute psychosocial stressor. Nx4 consists of three herbal extracts (*Passiflora incarnata, Avena sativa, Coffea arabica*) and a mineral salt (Zincum isovalerianicum) in low but ponderable concentrations. Nx4 has been previously reported to alter cortical electroencephalographic (EEG) spectral windows in rats and humans (Dimpfel, 2019; Dimpfel, Roeska, & Seilheimer, 2012), reduce stress‐induced increased amygdala activity as assessed by functional magnetic resonance imaging (fMRI; Herrmann et al., 2020), decrease human stress biomarkers following social performance stressors (Doering et al., 2016) and is implicated in calming restlessness/nervousness, and other symptoms associated with stress (Hübner, van Haselen, & Klein, 2009). Nx4 also improved self‐reported sleep latency and sleep disturbances in individuals with onset insomnia (Waldschütz & Klein, 2008).

In the present study, we administered Nx4 orally to rats and examined the sleep–wake EEG phenotypes in the context of the male rat dirty cage exchange method (CEX) as reported in Cano, Mochizuki, and Saper (2008). We selected the CEX model as it avoids potential confounds of a forced sleep deprivation protocol (Oonk, Krueger, & Davis, 2016), the neurocircuitry and electrophysiological correlates are characterised, and other rodent models of insomnia fall short of convincingly representing human insomnia conditions (Revel, Gottowik, Gatti, Wettstein, & Moreau, 2009). Cano et al. (2008) demonstrated an initial stress‐induced wakefulness (referred to as acute initial insomnia hereafter) that occurred in the first 2 h after exchange of soiled cages, followed by a 2 h period of no arousal state changes, which then transitioned to a delayed period of insomnia occurring 5–6 h after cage exchange. The initial insomnia may be particularly important to the diagnosis and treatment of patients with stress‐related onset insomnia, while the latter emergence of sleep disturbances described in the prefatorial CEX reports approximates that of patients with acute stress‐induced maintenance insomnia.

Other laboratories have exploited the male cage exchange model in conjunction with neurochemical profiling, rodent tinnitus, memory consolidation or preclinical drug testing, but this is the first documented use combining cortical EEG recordings with oral gavage in rats to administer drugs at the time of a single cage change (Gamble, Katsuki, McCoy, Strecker, & McKenna, 2020; Gvilia, Suntsova, Kumar, McGinty, & Szymusiak, 2015; Lee et al., 2016; McKenna et al., 2019; Yun et al., 2017; Zheng, Stiles, Chien, Darlington, & Smith, 2014). Based on previous findings that Nx4 positively affects brain activity of stress‐related areas, stress biomarkers, nervousness/restlessness, and insomnia, we hypothesised that administration of Nx4 prior to psychosocial stressor exposure would ameliorate the resulting disrupted sleep–wake patterns in rats.

## 2 METHODS

### 2.1 Animals

**FIGURE 1:**
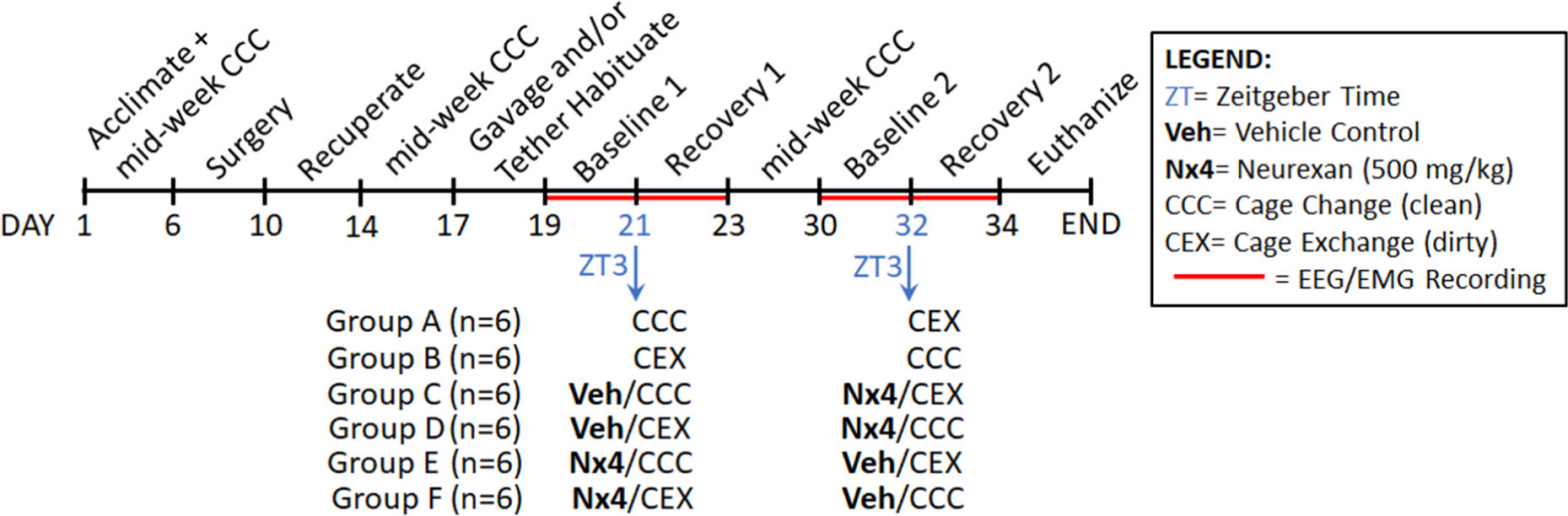
Experimental design and time course for testing Neurexan in the male cage exchange model

Male Sprague‐Dawley rats (aged 9–11 weeks at the time of surgery) procured from Envigo were transferred to a holding room on a 12:12 light/dark cycle with ambient temperatures of 24°C ± 2°C and allowed to acclimate for 6 days prior to the experiments. Rats were individually housed and had unrestricted access to food and water. Cages were also enriched with bedding materials and a Nylabone chew. Each rat was randomly assigned to a treatment group as part of a crossover design to control for possible order effects of cage change condition, clean or dirty (clean cage change [CCC] versus CEX; Figure 1). All animal procedures were approved by Washington State University’s Institutional Animal Care and Use Committee (IACUC; ASAF #6804) and were compliant with National Institutes of Health (NIH) guidelines. At the end of the study rats were euthanised by CO_2_ inhalation in a manner compliant with the *Panel on Euthanasia of the American Veterinary Medical Association*.

### 2.2 Surgery

The rats were surgically prepared using the methods described in Davis et al., 2011; Davis, Clinton, & Krueger, 2012, and Oonk et al., 2016. Briefly, rats were anaesthetised with intramuscular ketamine‐xylazine (87 and 13 mg/kg, respectively) and provided stainless steel screw EEG electrodes over the left parietal and frontal cortices positioned ~2 mm rostral or 5 mm caudal to the coronal suture and 3.0 mm left of the sagittal suture. An electromyography (EMG) electrode was placed in the dorsal neck muscles, and a stainless‐steel screw electrode affixed over the cerebellum 3.0 mm posterior to lambda served as an EMG ground. All wire leads were inserted in to a six‐pin plug interface (PlasticsOne) and secured to the skull with dental cement. After surgery the rats were given an analgesic injection of buprenorphine‐SR (extended‐release formula).

### 2.3 Gavage and tether/chamber habituation

After 4 days of recovery from surgery, the rats in the vehicle (Veh) and Nx4 treatment groups received an oral gavage of saline (0.9%) at zeitgeber time 3 (ZT3) for 3 days to reduce procedural stress of oral gavage in instrumented rats. The rats in all treatment groups were habituated to the recording chambers and tethered to a shielded biopotential cable connected to a six‐channel single‐brush commutator (PlasticsOne) for 2 days, after which baseline EEG/EMG recordings were initiated for 48 h.

### 2.4 EEG/EMG recording and arousal state determination

The analogue biopotential signals were sampled at 256 Hz, amplified (Grass 15LT) and filtered at 0.1 and 100 Hz with a notch filter of 60 Hz (Link 15 software). Filtered signals were collected using Vital Recorder software (Kissei Comtec) and digitised (A/D board; National Instruments) on a XPS i7 CPU (Dell) for subsequent analyses. File names were de‐identified for treatment through the assignment of non‐rapid eye movement sleep (NREM), REM, or wake state, files were then sorted based on treatment for further analysis. The NREM, REM, and waking vigilance states were determined manually off‐line in 10‐s epochs by trained technicians using SleepSign software (Kissei Comtec). NREM sleep was identified by high‐amplitude EEG signals and low EMG activity. Regular low‐amplitude EEG and minimal EMG activity characterised REM sleep. Wake periods were recognised by low amplitude fast EEG and high amplitude EMG activity.

### 2.5 Artefact exclusion

Identifying artefacts within rodent sleep recording is essential for establishing EEG spectral characteristics of distinct arousal states. The following method describes our seven‐step approach to performing this task using statistical thresholding before EEG spectral analyses.

1. Raw EEG data from all scored recordings of a single rat were loaded into the MATLAB workspace.
2. Any 10‐s epoch with an absolute maximum >95% (4.75 V) of the amplifiers’ physical cut‐off was marked as an artefact.
3. For each remaining epoch in all files associated with a single rat, the absolute maximum of the signal was extracted. A *z* score of this measure served as the first feature for each epoch.
4. A power spectral density was calculated for each remaining epoch using the Thomson multi‐taper method in 0.5‐Hz bins from 0.5 to 50 Hz. A *z* score for each 0.5‐Hz bin was then calculated across all epochs in all scored files for each rat. The mean of the resulting *z* score across all frequency bins served as the second feature for each epoch.
5. A threshold of >4 or <−2 was applied to the first feature, and a threshold of >2 or <−1 is applied to the second feature. Any epoch outside these thresholds was marked as artefact.
6. Steps 3–5 were repeated, re‐calculating features while excluding epochs that were previously marked as artefact, until no more artefacts were detected.
7. Steps 1–6 were performed for each rat in the study and the percentage of epochs excluded due to artefacts was then calculated for each rat’s entire record.

None of the rats had artefact‐excluded epochs that were >4% of the total recorded signal.

### 2.6 Experimental design

Before and after surgery the rats received clean cage exchanges twice a week (Figure 1). All rats were habituated to the recording chambers and tethered to a biopotential cable for 2 days (Day 17–18) after which baseline EEG/EMG recordings ensued for 48 h (Day 19–20). At ZT3 on day 21, rats in Group A (*n* = 6) were placed into a clean cage (CCC) and rats in Group B (*n* = 6) were placed into a soiled cage that had been inhabited for 1 week by a different male rat (CEX) and recordings continued for 48 h. After a 1‐week washout period, the experiment was repeated (Day 30–34) using a counterbalanced research design, such that Group A received CEX, and Group B received CCC. The same design was employed for examining Veh and Nx4, except that the rats in Groups C–F received gavage habituation (Day 17–19) and that prior to the cage change at ZT3 (Day 21), Group C rats (*n* = 6) were subjected to oral gavage of Veh and placed into a clean cage and Group D rats (*n* = 6) in a dirty cage. Recordings continued for 48 h (Day 21–22) and after a 1‐week washout the baseline recording was re‐established (Day 30–32), and at ZT3 on Day 32, Nx4 was oral gavaged and Group C rats were given CEX, and Group D rats were provided CCC followed by 48 h of additional recording (Day 32–34). The same research design and EEG/EMG recording schedule was then repeated with separate cohorts of rats that were initially administered Nx4 at ZT3 (Day 21) and subjected to CCC (Group E; *n* = 6) or CEX (Group F; *n* = 6) and Veh (Day 32) followed by exposure to the cage change condition that was different from the previous week. Thus, every rat in Groups C–F, received each experimental manipulation (viz. Veh, Nx4, CCC, and CEX) only once.

### 2.7 Study medications and dosing

The Nx4 tablets were manufactured by Heel GmbH Germany according to the German Regulation and international Good Manufacturing Practice (GMP) standards. The study medication was packaged, shipped, and labelled by Heel GmbH, Germany. The ingredients of Nx4 are listed in Table S1. Excipients include lactose monohydrate and magnesium stearate. The Veh tablets were composed of lactose monohydrate and magnesium stearate. Importantly, researcher blinding was accomplished with numerical de‐identification of Veh and Nx4 tablets throughout the entire term of the contract, medication delivery, and were not decoded until all data were analysed.

Study medications (Veh or Nx4 [500 mg/kg]) were given by oral gavage to rats in the Groups C–F, whereas rats assigned to Groups A and B received no oral gavage. The gavage solutions of Veh or Nx4 were prepared on the day of administration by crushing one tablet (~300 mg) and dissolving the powder in 1 ml of saline (0.9%). The appropriate gavage volume was determined using the weight of each rat such that a 300 g rat was given 150 mg of Veh or Nx4 in 0.5 ml of solution. Once drawn up in the syringe, the solution was inverted multiple times before the dose was administered to keep the suspension homogeneous.

### 2.8 Statistical analyses and outcome variables

Treatment order was analysed for each outcome measure and no order effects were found to be statistically significant. Therefore, pre‐ and post‐washout data were combined in the following six groups for comparison: CCC (*n* = 12), CEX (*n* = 12), Veh CCC (*n* = 11), Veh CEX (*n* = 11), Nx4 CCC (*n* = 11), and Nx4 CEX (*n* = 11) using the SPSS statistical package (IBM, version 17). One rat in Group D lost a headcap and one rat in Group F developed a malocclusion and both were excluded from the analysis. Time and frequency were analysed as repeated measures factors and treatment was analysed as a between subjects factor. A Greenhouse–Geisser correction on the degrees of freedom was made for sphericity violations on repeated measures, as determined by a Mauchley’s test, but the original degrees of freedom are reported for clarity. Where appropriate, post hoc Tukey’s or Fisher’s least significant difference tests were employed to determine differences between treatment groups (i.e., CCC, CEX, Veh CCC, Veh CEX, Nx4 CCC, and Nx4 CEX). Alpha was set at *p* < 0.05, while *p* ≥ 0.05 and ≤ 0.09 was interpreted as a non‐significant trend.

Sleep latency was defined as the amount of time (min) from tether reconnection after cage change to 1 min of consolidated NREM sleep. A one‐way analysis of variance (ANOVA) was used to compare treatment means for no gavage (*n* = 12/group) and gavaged rats (*n* = 11/group). The differences from baseline values in 2‐h time bins were used to analyse 0–24 h post‐cage change and 25–48 h post‐cage change (data not shown), for the time in each state (wake, NREM and REM sleep), state‐specific episode duration (as the average length of a wake, NREM or REM episodes in each 2‐h bin), and state‐specific episode frequency (as the number of wake, NREM or REM episodes in each 2‐h bin), using mixed two‐way ANOVAs with factors being time (repeated) and treatment group (between).

The delayed insomnia effects of CEX reported in Cano et al. (2008) were not observed (Figures S1‐S3) and the primary treatment effects occurred in the first 2 h after treatment. Furthermore, Cano et al. (2008) provide a priori justification for examining this time‐point as the initial stress response phase. Thus, one‐way between subjects’ ANOVAs of the first 2‐h post‐cage change compared each of the outcome measures, also expressed as the difference from baseline values.

After signal artefact removal, state‐specific fast Fourier transform (FFT) spectral power for NREM slow‐wave activity (SWA; 0.5–4 Hz), NREM gamma (30–60 Hz) and REM theta (5–9 Hz) were extracted in 2‐h time bins in SleepSign using a Hanning window. The FFT data from each rat were normalised to its averaged 2‐day baseline values and analysed using a two‐way ANOVA of time (repeated) and treatment group (between). Then, based on the NREM SWA, gamma and REM theta datasets we selected post‐cage change hours 2–4 to run state‐specific EEG spectral analyses in the 0–60 Hz range in 1‐Hz bins. We utilised the fifth iteration of the auto‐data imputation algorithm (SPSS) to supplant time bins with missing data values for repeated measures. Mixed two‐way ANOVAs with Hz (repeated) and treatment group (between) factors were used to compare across conditions.

## 3 RESULTS

Despite the claim of CEX being a model platform to examine psychosocial stress‐related insomnia in rats, key findings of Cano et al. (2008) are challenged herein, namely the lack of the delayed stress‐induced insomnia at the 5–6 h post‐cage change period as demonstrated by an absence of changes in sleep and wake architecture or increase in NREM EEG gamma activity (Figures S1‐S4). Our dataset is not alone in the inability to replicate the delayed phase insomnia findings (McKenna et al., 2019; Zheng et al., 2014).

### 3.1 Exposure to a dirty cage induces an initial insomnia

**FIGURE 2:**
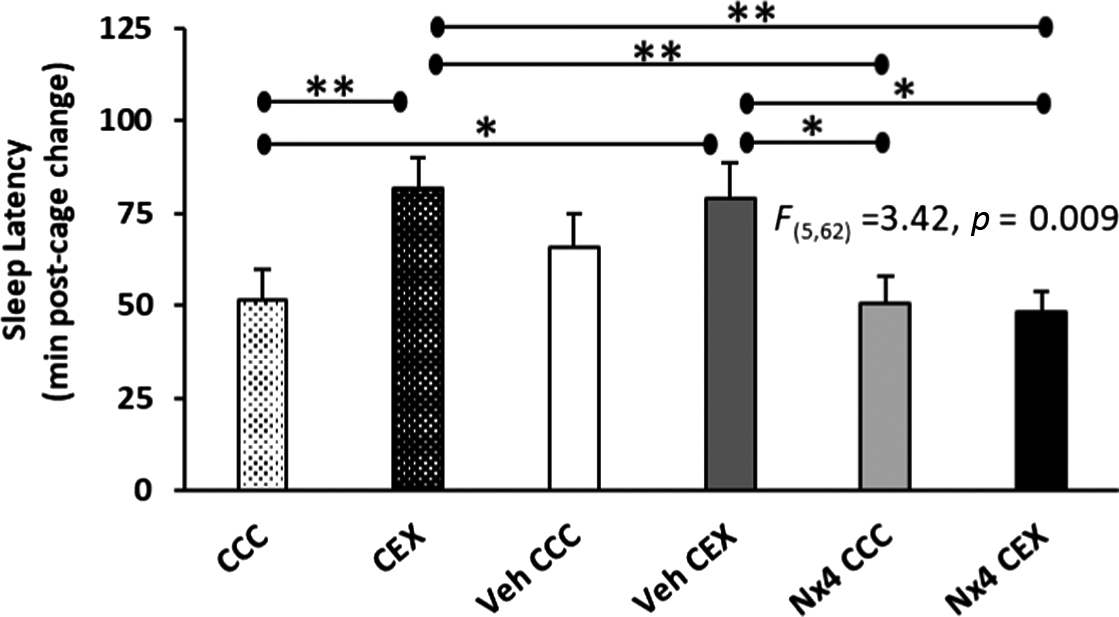
Post‐cage change sleep latency data presented as mean ± standard errors. Data were statistically analysed using a one‐way analysis of variance (ANOVA), *F*‐statistic indicated. CCC, clean cage change; CEX, dirty cage exchange; Veh, vehicle control; Nx4, Neurexan; *p < 0.05; **p < 0.01. CCC and CEX, *n* = 12; Veh CCC, Veh CEX, Nx4 CCC and Nx4 CEX, *n* = 11

Consistent with the Cano et al. (2008) findings, we report that post‐cage change NREM sleep latency in CEX rats was increased by 30.42 min compared to rats in the CCC group (*p* = 0.008, Figure 2). This suggests a psychosocial stressor‐induced initial insomnia, sometimes referred to as the initial stress response or stress‐induced hyperarousal (Bonaventure et al., 2015; Cano et al., 2008). This insomnia phenotype evidenced in the CEX model may be particularly important to the diagnosis and treatment of patients with acute stress‐related onset insomnia.

**FIGURE 3:**
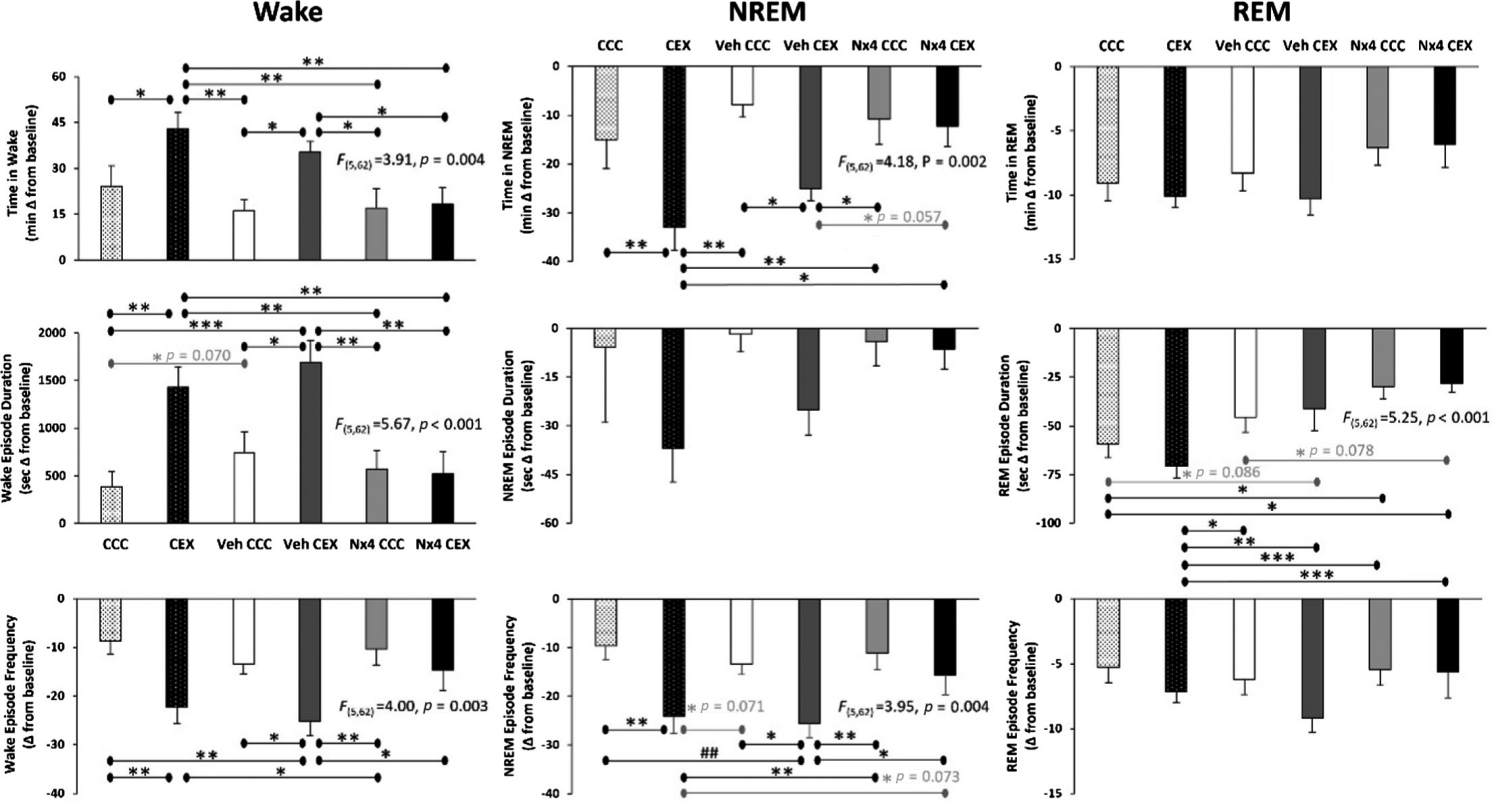
Wake (left column), NREM (centre column) and REM (right column) state‐specific EEG phenotypes for 0–2 h after cage change data presented as mean difference from baseline ± standard error. Baseline EEG values were collected from time‐matched averages collected on the 2 days preceding cage change. Data were statistically analysed using one‐way analyses of variance (ANOVAs), statistically significant *F*‐statistics indicated. NREM, non‐rapid eye movement sleep; REM, rapid eye movement sleep; EEG, electroencephalographic; CCC, clean cage change; CEX, dirty cage exchange; Veh, vehicle control; Nx4, Neurexan. *p < 0.05; **p < 0.01; ***p < 0.001. Grey font = trend with *p*‐value indicated. CCC and CEX, *n* = 12; Veh CCC, Veh CEX, Nx4 CCC and Nx4 CEX, *n* = 11

The acute initial insomnia was also evidenced by 19.0 min of additional time awake (*p* = 0.014), 17.4 min longer wake episode duration (*p* = 0.001), and 13.6 fewer wake episodes (*p* = 0.004) over the first 2 h after dirty cage exposure compared to rats that were transferred to a previously uninhabited clean cage (Figure 3, left panels). CEX rats also manifest concurrent decreases in NREM sleep by 18.0 min (*p* = 0.006) and 14.5 fewer NREM sleep episodes (*p* = 0.002) than the CCC rats (Figure 3, centre panels), but REM sleep differences between CCC and CEX rats were not statistically significant (Figure 3, right panels). Together these findings demonstrate the utility of the CEX model for eliciting acute sleep disturbances resulting from exposure to a psychosocial stressor.

### 3.2 Nx4 mitigates acute stress‐induced initial insomnia

**FIGURE 4:**
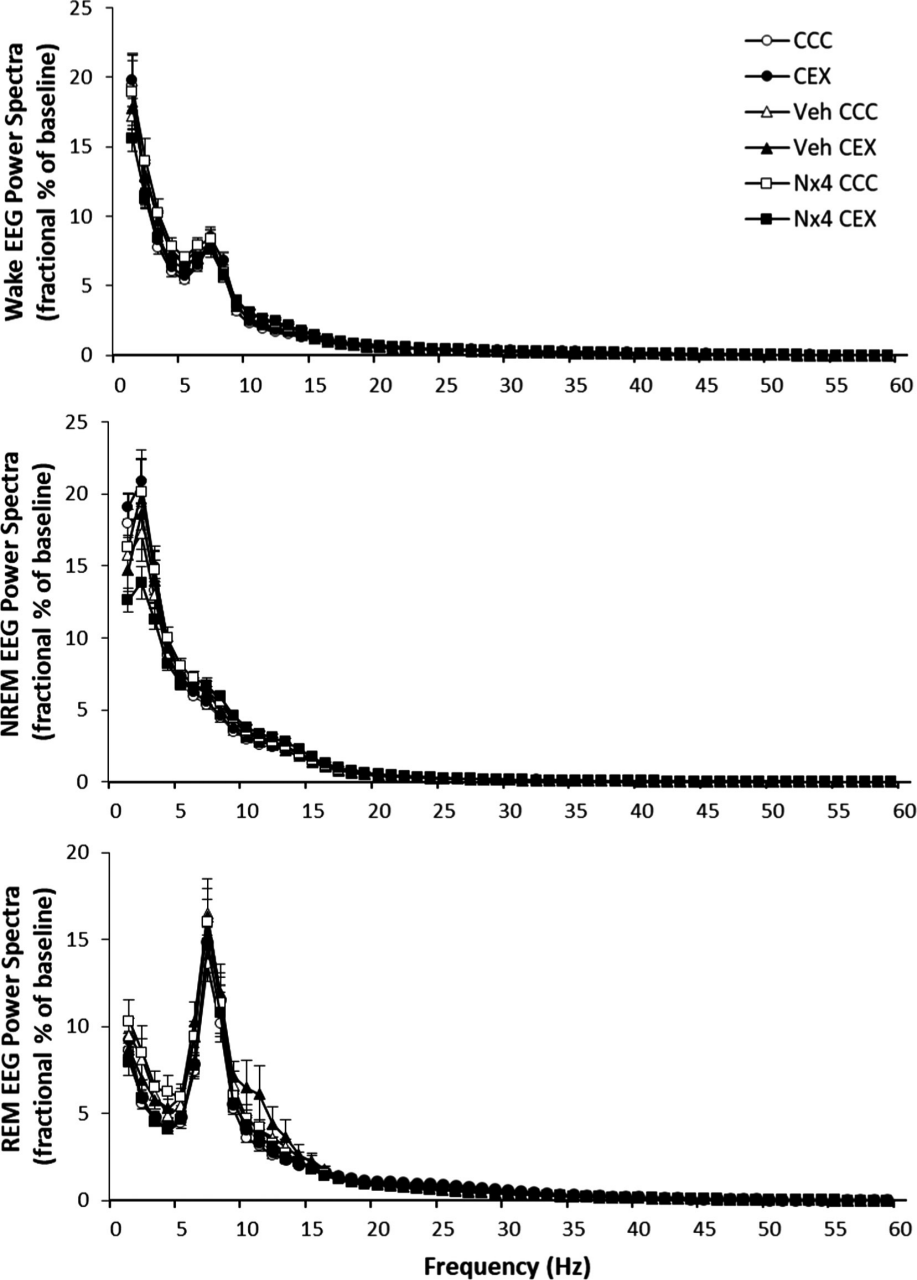
Wake, NREM and REM state‐specific EEG power spectra 2–4 h after cage change data presented as mean ± standard error. Baseline EEG values were collected from time‐matched averages of the 2 days preceding cage change. Data were statistically analysed using mixed two‐way analyses of variance (ANOVAs). NREM, non‐rapid eye movement sleep; REM, rapid eye movement sleep; EEG, electroencephalographic; CCC, clean cage change; CEX, dirty cage exchange; Veh, vehicle control; Nx4, Neurexan. CCC and CEX, *n* = 12; Veh CCC, Veh CEX, Nx4 CCC and Nx4 CEX, *n* = 11

A primary finding reported herein is the somnogenic effects of Nx4 in mitigating acute stress‐induced initial insomnia. First, Nx4 CEX and Nx4 CCC rat averages were generally quite similar, and no sleep–wake phenotypes were statistically different (Figures 2, 3, 4). This result was further illustrated by significant differences detected between Nx4 CEX rats and CEX and/or Veh CEX rats on sleep latency, time awake, wake episode duration and frequency, and NREM episode frequency outcome measures. Specifically, Nx4 CEX rats fell asleep 33.3 min before CEX rats (*p* = 0.005) and 30.4 min before Veh CEX rats (*p* = 0.011, Figure 2). The magnitude of the Nx4 effect on sleep latency exceeds those reported using orexinergic antagonist hypnotics tested with a CEX model (Bonaventure et al., 2015; Gamble et al., 2020; Yun et al., 2017). Sleep latency is correlated with sleep–wake duration and other arousal metrics, especially when the analysis time bins are short. In the first 2 h after cage change, Nx4 CEX rats were awake 24.7 and 16.9 min less than CEX and Veh CEX rats, respectively (*p* = 0.002 and *p* = 0.034, respectively, Figure 3, left panels). The Nx4 CEX rats had 15.2 min shorter wake episodes compared with CEX rats (*p* = 0.005) and 19.6 min shorter (*p* = 0.001) and 10.6 more (*p* = 0.028) wake episodes than Veh CEX for the first 2 h after cage change and thereby Nx4 restored the number of wake episodes to approximately control levels. Furthermore, Nx4 CEX rats had 20.6 min more NREM sleep than CEX rats after cage change (*p* = 0.002, Figure 3, centre panels). In addition, there was also a strong trend (*p* = 0.057) with Nx4 CEX rats spending 12.7 min more time in NREM sleep than Veh CEX. The Nx4 CEX rats had 10.0 more NREM episodes compared to Veh CEX rats (*p* = 0.041) and had 8.5 more NREM episodes than CEX rats, but the latter difference was a trend (*p* = 0.073). Time in REM sleep and REM episode frequency were not statistically significant between groups; however, the length of REM episodes in Nx4 CEX rats averaged 42.7 s longer than in CEX rats (*p* < 0.001; Figure 3, right panels). Finally, no statistically significant changes between any treatment groups were detected in the state specific EEG spectral windows analysed including NREM delta, NREM gamma or REM theta activity (Figures 4 and S4), although trends were detected in NREM delta (*p* = 0.070) and gamma (time × treatment interaction, *p* = 0.071).

### 3.3 Procedural stress may impact the CEX model

To our knowledge this is the only study combining oral gavage restraint and EEG/EMG headstage‐instrumented rats using the single CEX model. The conflated effects of gavage in headstage‐equipped rats likely constitutes a procedural stressor. This approach may have altered the EEG results in the first 2‐h post‐cage change period even after subjecting rats to a gavage habituation protocol prior to recording. Several findings point to a procedural stressor that may have slightly impacted the CEX model. First, Veh CCC rats fell asleep 13.2 min quicker post‐cage change than Veh CEX rats (*p* = 0.261, Figure 2). This is less than half of the difference between the non‐gavaged CCC and CEX rats and was therefore not statistically significant. Secondly, we anticipated that Veh CEX rats would differ from CCC rats on sleep–wake amounts, as we observed with NREM sleep and wake episode frequency and with wake episode duration, but these comparisons were not statistically significant (Figure 3, top row). Likewise, we expected that CEX rats would differ from Veh CCC rats in wake episode duration and sleep–wake frequency, as was observed with time in wake or NREM sleep. Other than REM episode duration, statistical significance was not reached between groups for REM data (Figure 3). However, REM sleep data should be interpreted with caution as generally REM sleep in rats has more between‐subject variability and is prone to under sampling by its lower occurrence compared to wake or NREM sleep vigilance states during the first 2 h of rats’ sleep. Regardless, the presence of a putative procedural stressor did not diminish the somnogenic effects of Nx4 reported herein as it was distributed across all four gavaged conditions. While it may have altered the variability in some rat outcome measures, Nx4 was still able to assuage CEX stress‐induced initial insomnia. Alternative routes of administration or telemetric EEG implants may be indicated with use of the CEX model to investigate the effects of novel compounds.

## 4 DISCUSSION

Overall, the present results support our hypothesis that Nx4 treatment demonstrates somnogenic effects that ameliorate acute psychosocial stressor‐induced initial insomnia. These data complement other published reports implicating the therapeutic value of Nx4. For example, Nx4 decreases stress‐induced increased cortical and amygdala activity as assessed by EEG and fMRI (Dimpfel, 2019; Herrmann et al., 2020). In preclinical work, Dimpfel et al. (2012) demonstrated altered EEG frequencies in rats that received Nx4 and showed significant effects predominantly on delta‐ and theta‐waves in the frontal cortex and reticular formation. This finding was interpreted as Nx4’s potential for producing calming effects. The present study did not yield significant changes in delta or theta changes, although NREM delta and gamma trended towards statistical significance (*p* = 0.070 and *p= 0.071, respectively*; Figure S4). This discrepancy could be explained by a key technical difference in biopotential sampling of local field potentials in Dimpfel et al. (2012) versus cortical surface EEGs. Moreover, changes in proximal cortical activity and local bioavailability of neurotransmitters could also explain the differences. Waldschütz and Klein (2008) report that Nx4 reduced sleep latency, improved sleep duration, and quality of sleep in patients with mild‐to‐moderate sleep disturbances. Clinical evidence also found that compared with placebo, Nx4 significantly reduced stress‐induced increases in stress biomarkers including, salivary cortisol and plasma adrenaline and showed a trend to diminish stress‐induced increase in plasma cortisol (Doering et al., 2016). Moreover, Hübner et al. (2009) provided evidence that Nx4 reduces symptoms typically associated with stress. Nx4 is also implicated in other stress‐related physiologies, e.g., Nx4 treatment was effective at increasing heart rate variability (Chand et al., 2019). Combined, these data suggest a potential benefit of Nx4 for individuals experiencing stress‐related sleep disturbances. And given that evidence of the stress‐associated aspect of acute insomnia was absent in prior studies of Nx4, the present results further specify a therapeutic advantage of the drug in such conditions. Whether one experiences difficulties with sleep onset or maintaining sleep, the compounding effects of stress and insufficient sleep can adversely affect mood and day‐time performance. Thus, Nx4 could increase nocturnal satisfaction with sleep and enhance daytime functioning by reducing fatigue, mood disturbances, and cognitive decrements over the day.

A primary objective of our investigation was to show that Nx4 ameliorated the acute stress‐induced initial and delayed insomnia of the Cano et al. (2008) study, but we were unable to reproduce several key findings, i.e., the delayed stress‐induced insomnia at the 5–6 h post‐cage change period, an increase in EEG gamma activity and changes in REM sleep although matching strain, sex, age, and reported protocol was achieved. While other studies have implemented the use of the CEX model to test hypnotic compounds or other phenomena, they were either unable to replicate core findings of the Cano study (McKenna et al., 2019; Zheng et al., 2014), or did not record EEG (Lee et al., 2016; Zheng et al., 2014), did not compare to a CCC (Bonaventure et al., 2015; Gamble et al., 2020; Gvilia et al., 2015; Lee et al., 2016; McKenna et al., 2019; Yun et al., 2017) or used a different species (Bonaventure et al., 2015; Yun et al., 2017) making it difficult to compare results. The search for a CEX‐induced delayed insomnia phase study was partly resolved by implementing two cage changes in succession with each cage soiled by a separate rat (Gamble et al., 2020; McKenna et al., 2019). They observed another transient insomnia at 4 h, which was the hour following the second cage change. However, the double dirty cage change technique could arguably represent two separate initial insomnias that can impede memory (Gamble et al., 2020; McKenna et al., 2019), but limits its validity to model maintenance insomnia. Like our present findings, McKenna et al. (2019 also failed to see the changes in REM sleep and NREM gamma activity was not reported.

## 5 CONCLUSION

In summary, the multicomponent drug Nx4 has somnogenic effects and overcomes disrupted sleep incurred by acute psychosocial stressor exposure in rats. This research can be regarded as translational, as rodent and human sleep architecture correlate well on many traits, and rodents and humans react to hypnotics in similar ways. Growing evidence indicates that the multicomponent composition appears to provide multitarget effects that are not isolated to sleep health. Future research needs to further elucidate the molecular effects of Nx4 on the networks related to sleep and the stress response.

## AUTHOR CONTRIBUTIONS

Christopher J. Davis – conceptualisation, data curation, formal analysis, investigation, methodology, resources, supervision, validation, visualisation, provided original draft, review and edit manuscript. Michelle A. Schmidt – data curation, investigation, methodology, review and edit manuscript. Kathrin Hemmer and Natascha Krömmelbein – conceptualisation, methodology, validation, project administration, writing – review and editing. Bernd Seilheimer – conceptualisation, funding acquisition, project administration, supervision, validation, writing – review and editing.

## CONFLICT OF INTERST

Following submission of the final report of the subcontract C.J.D. was compensated for manuscript editing by Heel GmbH. K.H. and B.S. are employed by Heel GmbH. N.K. was employed by Heel GmbH at an early stage of study implementation.

## Supporting information

Fig S1‐S4

Table S1

## Data Availability

The data that support the findings of this study are available from the corresponding author upon reasonable request.

## References

[jsr13550-bib-0001] Åkerstedt, T. , Nordin, M. , Alfredsson, L. , Westerholm, P. , & Kecklund, G. (2012). Predicting changes in sleep complaints from baseline values and changes in work demands, work control, and work preoccupation–the WOLF‐project. Sleep Medicine, 13, 73–80. 10.1016/j.sleep.2011.04.015 22177346

[jsr13550-bib-0002] Blanco‐Encomienda, F.J. , García‐Cantero, R. , & Latorre‐Medina, M.J. (2020). Association between Work‐related rumination, work environment and employee well‐being: A meta‐analytic study of main and moderator effects. Social Indicators Research, 150, 887–910. 10.1007/s11205-020-02356-1

[jsr13550-bib-0003] Bonaventure, P. , Yun, S. , Johnson, P.L. , Shekhar, A. , Fitz, S.D. , Shireman, B.T. , … Dugovic, C. (2015). A selective orexin‐1 receptor antagonist attenuates stress‐induced hyperarousal without hypnotic effects. The Journal of Pharmacology and Experimental Therapeutics, 352(3), 590–601. 10.1124/jpet.114.220392 25583879 PMC4352589

[jsr13550-bib-0004] Cano, G. , Mochizuki, T. , & Saper, C.B. (2008). Neural circuitry of stress‐induced insomnia in rats. Journal of Neuroscience, 28, 10167–10184. 10.1523/JNEUROSCI.1809-08.2008 18829974 PMC2693213

[jsr13550-bib-0005] Chand, T. , Jamalabadi, H. , Alizadeh, S. , Sen, Z.D. , Fensky, L. , & Walter, M. (2019). Effects of Nx4 on reduced stress responsibility reflected by heart rate variability. Psychoneuroendocrinology, 107, 12. 10.1016/j.psyneuen.2019.07.032

[jsr13550-bib-0006] Davis, C.J. , Clinton, J.M. , & Krueger, J.M. (2012). MicroRNA 138, let‐7b, and 125a inhibitors differentially alter sleep and EEG delta‐wave activity in rats. Journal of Applied Physiology, 113, 1756–1762. 10.1152/japplphysiol.00940.2012 23104698 PMC3544506

[jsr13550-bib-0007] Davis, C.J. , Clinton, J.M. , Taishi, P. , Bohnet, S.G. , Honn, K.A. , & Krueger, J.M. (2011). MicroRNA 132 alters sleep and varies with time in brain. Journal of Applied Physiology, 111, 665–672. 10.1152/japplphysiol.00517.2011 21719725 PMC3174793

[jsr13550-bib-0008] Dimpfel, W. (2019). Effects of neurexan on stress‐induced changes of spectral EEG power: A double‐blind, randomized, placebo‐controlled, crossover exploratory trial in human volunteers. World Journal of Neuroscience, 09, 100–112. 10.4236/wjns.2019.93007

[jsr13550-bib-0009] Dimpfel, W. , Roeska, K. , & Seilheimer, B. (2012). Effect of Neurexan on the pattern of EEG frequencies in rats. BMC Complementary and Alternative Medicine, 12, 126. 10.1186/1472-6882-12-126 22898322 PMC3493358

[jsr13550-bib-0010] Doering, B.K. , Wegner, A. , Hadamitzky, M. , Engler, H. , Rief, W. , & Schedlowski, M. (2016). Effects of Neurexan ^®^ in an experimental acute stress setting — An explorative double‐blind study in healthy volunteers. Life Sciences, 146, 139–147. 10.1016/j.lfs.2015.12.058 26772822

[jsr13550-bib-0011] Ferrie, J.E. , Kumari, M. , Salo, P. , Singh‐Manoux, A. , & Kivimäki, M. (2011). Sleep epidemiology – a rapidly growing field. International Journal of Epidemiology, 40(6), 1431–1437. 10.1093/ije/dyr203 22158659 PMC3655374

[jsr13550-bib-0012] Gamble, M.C. , Katsuki, F. , McCoy, J.G. , Strecker, R.E. , & McKenna, J.T. (2020). The dual orexinergic receptor antagonist DORA‐22 improves the sleep disruption and memory impairment produced by a rodent insomnia model. Sleep, 43(3), zsz241. 10.1093/sleep/zsz241 31595304

[jsr13550-bib-0013] Garefelt, J. , Platts, L.G. , Hyde, M. , Magnusson Hanson, L.L. , Westerlund, H. , & Åkerstedt, T. (2020). Reciprocal relations between work stress and insomnia symptoms: A prospective study. Journal of Sleep Research, 29, e12949. 10.1111/jsr.12949 31793085 PMC7154699

[jsr13550-bib-0014] Gvilia, I. , Suntsova, N. , Kumar, S. , McGinty, D. , & Szymusiak, R. (2015). Suppression of preoptic sleep‐regulatory neuronal activity during corticotropin‐releasing factor‐induced sleep disturbance. American Journal of Physiology. Regulatory, Integrative and Comparative Physiology, 309(9), R1092–R1100. 10.1152/ajpregu.00176.2015 26333784

[jsr13550-bib-0015] Herrmann, L. , Vicheva, P. , Kasties, V. , Danyeli, L. , Szycik, G. , Denzel, D. , … Walter, M. (2020). fMRI revealed reduced amygdala activation after Nx4 in mildly to moderately stressed healthy volunteers in a randomized, placebo‐controlled, cross‐over trial. Scientific Reports, 10, 1–14. 10.1038/s41598-020-60392-w 32123197 PMC7052227

[jsr13550-bib-0016] Hübner, R. , van Haselen, R. , & Klein, P. (2009). Effectiveness of the homeopathic preparation Neurexan compared with that of commonly used valerian‐based preparations for the treatment of nervousness/restlessness: an observational study. Scientific World Journal, 9, 733–745. 10.1100/tsw.2009.95 19705035 PMC5823074

[jsr13550-bib-0017] Lee, D.‐W. , Chung, S. , Yoo, H.J. , Kim, S.J. , Woo, C.‐W. , Kim, S.‐T. , … Woo, D.‐C. (2016). Neurochemical changes associated with stress‐induced sleep disturbance in rats. In vivo and in vitro measurements. PLoS One, 11(4), e0153346. 10.1371/journal.pone.0153346 27078855 PMC4831675

[jsr13550-bib-0018] Magnusson Hanson, L. , Åkerstedt, T. , Näswall, K. , Leineweber, C. , Theorell, T. , & Westerlund, H. (2011). Cross‐lagged relationships between workplace demands, control, support, and sleep problems. Sleep, 34(10), 1403–1410. 10.5665/SLEEP.1288 21966072 PMC3174842

[jsr13550-bib-0019] McKenna, J.T. , Gamble, M.C. , Anderson‐Chernishof, M.B. , Shah, S.R. , McCoy, J.G. , & Strecker, R.E. (2019). A rodent cage change insomnia model disrupts memory consolidation. Journal of Sleep Research, 28(2), e12792. 10.1111/jsr.12792 30461100

[jsr13550-bib-0020] Oonk, M. , Krueger, J.M. , & Davis, C.J. (2016). Voluntary sleep loss in rats. Sleep, 39, 1467–1479. 10.5665/sleep.5984 27166236 PMC4909628

[jsr13550-bib-0021] Revel, F.G. , Gottowik, J. , Gatti, S. , Wettstein, J.G. , & Moreau, J.L. (2009). Rodent models of insomnia: a review of experimental procedures that induce sleep disturbances. Neuroscience and Biobehavioral Reviews, 33(6), 874–899. 10.1016/j.neubiorev.2009.03.002 19428498

[jsr13550-bib-0022] Waldschütz, R. , & Klein, P. (2008). The homeopathic preparation Neurexan vs. valerian for the treatment of insomnia: an observational study. Scientific World Journal, 8, 411–420. 10.1100/tsw.2008.61 18454251 PMC5848849

[jsr13550-bib-0023] Yun, S. , Wennerholm, M. , Shelton, J.E. , Bonaventure, P. , Letavic, M.A. , Shireman, B.T. , … Dugovic, C. (2017). Selective inhibition of orexin‐2 receptors prevents stress‐induced ACTH release in mice. Frontiers in Behavioral Neuroscience, 11, 83. 10.3389/fnbeh.2017.00083 28533747 PMC5420581

[jsr13550-bib-0024] Zheng, Y. , Stiles, L. , Chien, Y.T. , Darlington, C.L. , & Smith, P.F. (2014). The effects of acute stress‐induced sleep disturbance on acoustic trauma‐induced tinnitus in rats. BioMed Research International, 2014, 1–8. 10.1155/2014/724195 PMC4137606 25162023

