# Supplementary material for: Multicomponent drug Neurexan mitigates acute stress‐induced insomnia in rats
Source: J Sleep Res. 2022 Jan 21;31(5):e13550. doi: 10.1111/jsr.13550 (PMC9786571; doi:10.1111/jsr.13550)

Supplemental Figure 1. Wake state EEG architecture 0-24 hours following cage change data presented as mean difference ± standard error. Baseline EEG values were collected from time-matched averages collected on the two days preceding cage change. Data were statistically analyzed using mixed two-way ANOVAs. EEG=electroencephalographic; CCC=clean cage change; CEX=dirty cage exchange; Veh= vehicle control; Nx4=Neurexan (CCC and CEX n=12; Veh CCC, Veh CEX, Nx4 CCC and Nx4 CEX n=11).


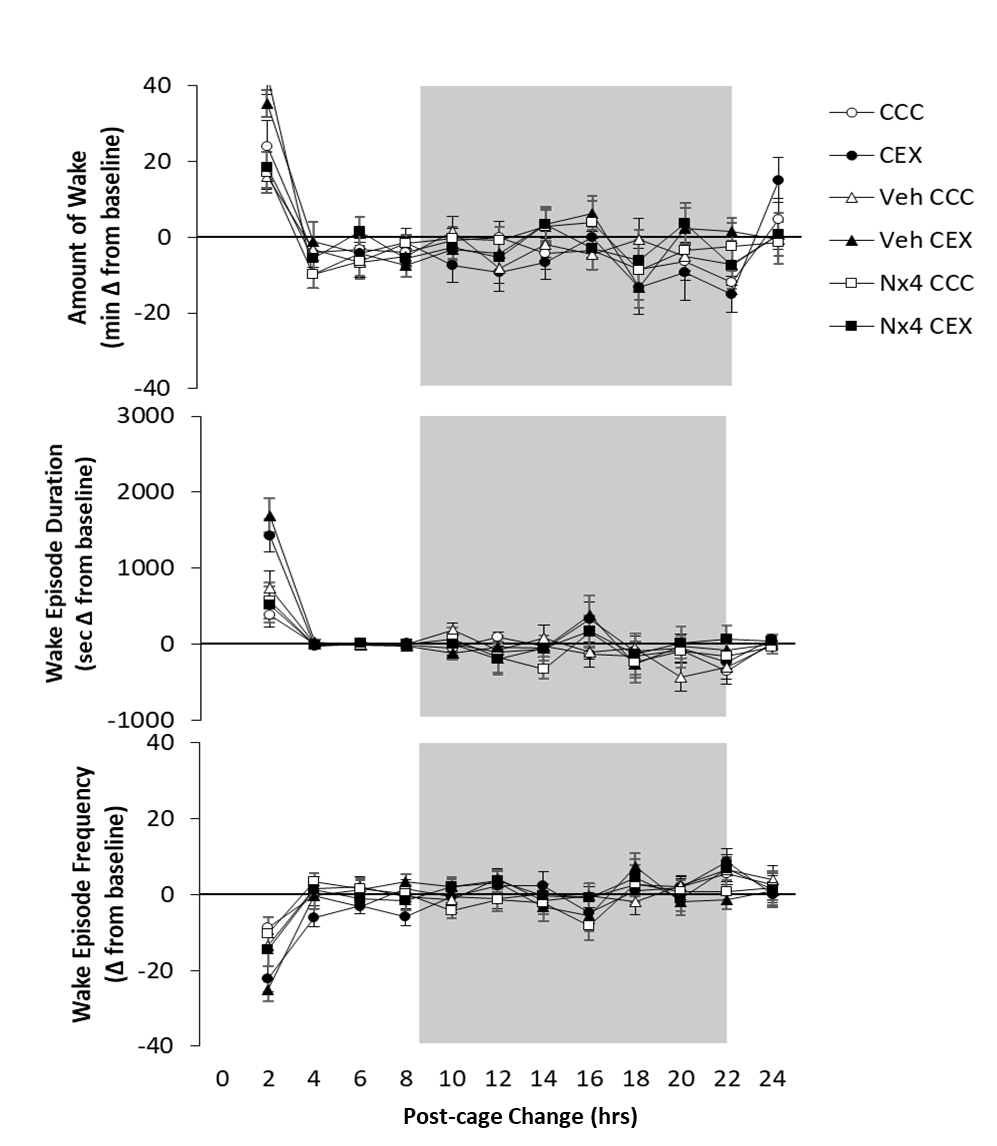


Supplemental Figure 2. NREM sleep state EEG architecture 0-24 hours following cage change data presented as mean difference ± standard error. Baseline EEG values were collected from time-matched averages collected on the two days preceding cage change. Data were statistically analyzed using mixed two-way ANOVAs. NREMS=non-rapid eye movement sleep; EEG=electroencephalographic; CCC=clean cage change; CEX=dirty cage exchange; Veh= vehicle control; Nx4=Neurexan (CCC and CEX n=12; Veh CCC, Veh CEX, Nx4 CCC and Nx4 CEX n=11).
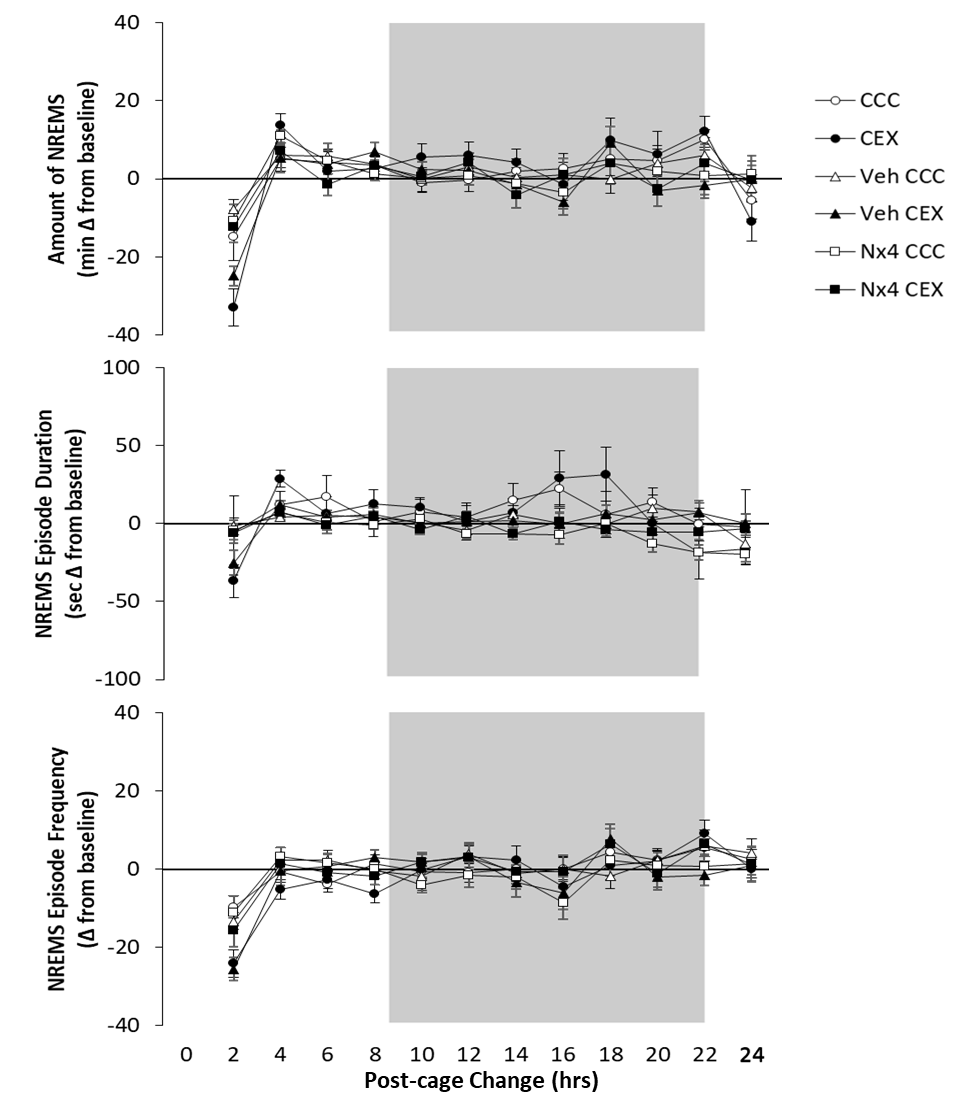


Supplemental Figure 3. REM sleep state architecture 0-24 hours following cage change data presented as mean difference ± standard error. Baseline EEG values were collected from time-matched averages collected on the two days preceding cage change. Data were statistically analyzed using mixed two-way ANOVAs. REMS=rapid eye movement sleep; EEG=electroencephalographic; CCC=clean cage change; CEX=dirty cage exchange; Veh= vehicle control; Nx4=Neurexan (CCC and CEX n=12; Veh CCC, Veh CEX, Nx4 CCC and Nx4 CEX n=11).


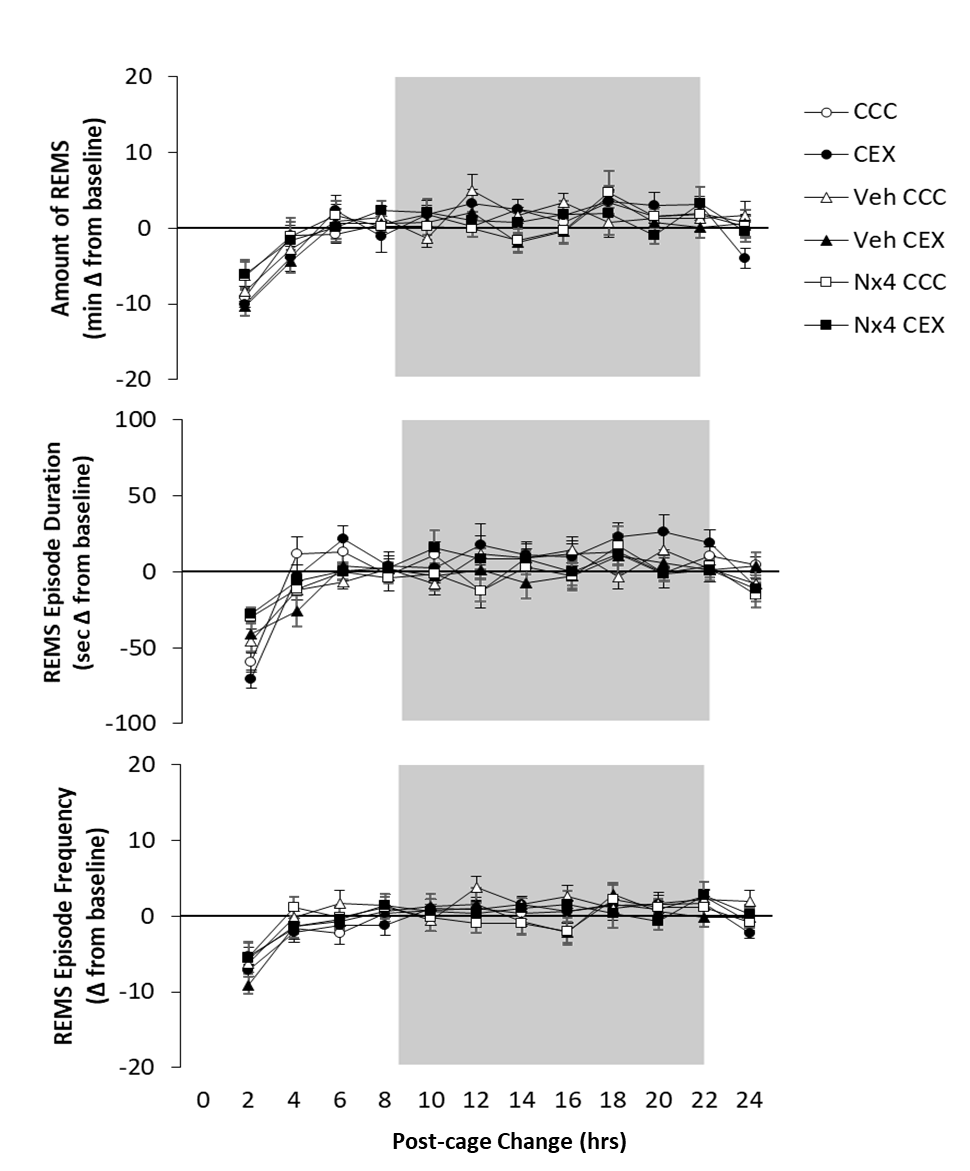


Supplemental Figure 4. State-specific spectral bands 0-24 hrs after cage change data presented as mean percent of baseline ± standard error. Baseline EEG values were collected from time-matched averages collected on the two days preceding cage change. Data were statistically analyzed using two-way ANOVAs, F-statistic meeting trend criterion indicated. NREMS=non-rapid eye movement sleep; REMS=rapid eye movement sleep; EEG=electroencephalographic; CCC=clean cage change; CEX=dirty cage exchange; Veh= vehicle control; Nx4=Neurexan (CCC and CEX n=12; Veh CCC, Veh CEX, Nx4 CCC and Nx4 CEX n=11).


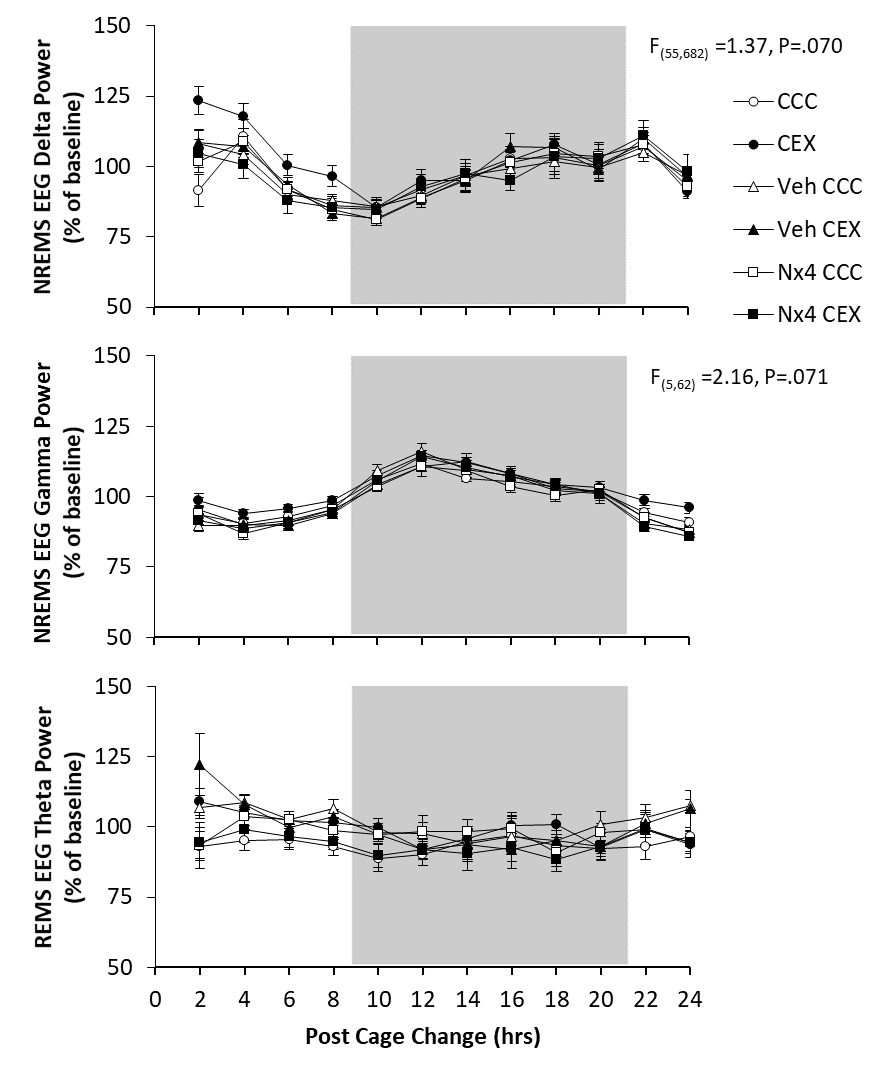

Supplement: Supplementary file 1 — Fig S1‐S4 [file JSR-31-e13550-s002.docx]
