# Supplementary material for: Multicomponent drug Neurexan mitigates acute stress‐induced insomnia in rats
Source: J Sleep Res. 2022 Jan 21;31(5):e13550. doi: 10.1111/jsr.13550 (PMC9786571; doi:10.1111/jsr.13550)
Supplement: Supplementary file 2 — Table S1 [file JSR-31-e13550-s001.docx]

Supplemental Table 1. Composition of Neurexan tablets.

| **Component** | | **Manufacturing method (Ph. Eur.)** | **µg/tablet in Neurexan** |
| --- | --- | --- | --- |
| *Plant extracts* | | | |
|  | Fresh aerial parts of *Avena sativa* L., collected at flowering time (Oat) | Method 1.1.1 | 12 |
|  | Ripe, dried, unroasted seeds of *Coffea arabica* L. with the seed coat (silver skin) largely removed (Arabian coffee) | Method 1.1.8 | 0.6 1E-8 |
|  | Fresh aerial parts of *Passiflora incarnata* L.  (Passionflower) | Method 1.1.5 | 18 |
| *Chemical substances* | | | |
|  | Zincum isovalerianicum | Method 4.1.1 | 0.06 |
|  |  |  |  |

Ph. Eur. = European Pharmacopoeia
